# Inappropriate treatment of hospital-acquired infections and associated factors among admitted adults in Wolaita Zone hospitals, Southern Ethiopia: A multi-center cross-sectional study

**DOI:** 10.1371/journal.pone.0339116

**Published:** 2025-12-26

**Authors:** Abera Abiye Jaguba, Bekan Alemu, Temesgen Leka Lerango, Hailu Chare Koyra

**Affiliations:** 1 School of Pharmacy, College of Health Sciences and Medicine, Wolaita Sodo University, Wolaita Sodo, Ethiopia; 2 School of Public Health, College of Health Sciences and Medicine, Dilla University, Dilla, Ethiopia; Hawassa University, ETHIOPIA

## Abstract

**Background:**

Hospital-acquired infections (HAIs) are a global health concern. Inappropriate treatment of HAIs worsens their impact, contributing to increased antimicrobial resistance, higher healthcare costs, and heightened morbidity and mortality. However, evidence on inappropriate treatment of HAIs in resource-constrained settings remains limited. The aim of this study was to assess the prevalence and associated factors of inappropriate treatment of HAIs among admitted adults in Wolaita Zone hospitals, Southern Ethiopia.

**Methods:**

A multicenter cross-sectional study was conducted in selected hospitals in the Wolaita Zone from 28 October 2024–25 February 2025, enrolling 280 patients with HAIs. Data were collected via structured face-to-face interviews and medical record review. Data were entered in EpiData 4.6 and analyzed in SPSS 25. A binary logistic regression model was employed to examine the associations between the outcome variable and explanatory variables, with statistical significance determined at a p-value < 0.05.

**Results:**

The mean (± SD) age of participants was 41.25 ± 12.5 years. Pneumonia was the most frequently diagnosed HAI (133, 47.5%). *Streptococcus* species, *Staphylococcus aureus*, and *Klebsiella pneumoniae* were the predominant pathogens isolated. The overall prevalence of inappropriate treatment of HAI was 53.6% (95% CI: 47.5–59.5). The odds of inappropriate treatment of HAI were significantly higher in the presence of comorbidity (AOR = 2.69, 95% CI: 1.45–5.01; *p = 0.002*) and among patients treated in the surgical ward (AOR = 2.75, 95% CI: 1.32–5.74; *p = 0.007*). Conversely, culture testing (AOR = 0.34, 95% CI: 0.15–0.74; *p = 0.007*) and provision of clinical pharmacy service (AOR = 0.28, 95% CI: 0.13–0.60; *p = 0.001*) were associated with reduced odds of inappropriate treatment of HAIs.

**Conclusions:**

The prevalence of inappropriate treatment of HAIs among admitted adults in the current study settings was high. Comorbidities, treatment ward type, culture testing, and the provision of clinical pharmacy services were significantly associated with inappropriate treatment. Clinicians should give particular attention to patients with comorbidities and those receiving care in surgical wards. Health facilities should enhance microbiological diagnostic services to promote evidence-based therapy. Additionally, clinical pharmacy services should be expanded across hospital wards to support efforts in reducing inappropriate treatment of HAIs. These findings underscore the urgent need for multifaceted interventions to improve patient care and combat antimicrobial resistance in this setting.

## Introduction

Hospital acquired infections (HAIs) are illnesses, either localized or systemic, that develop as a reaction to an infectious agent, bacteria, or its toxins that were present in hospital settings but were not incubating or exhibiting symptoms when the patient was admitted to the hospital [1,2]. These infections are usually of nosocomial origin and typically manifest clinically at least 48 hours after hospital admission or within 30 days after having received health care [3,4]. The most common HAIs include surgical site infection (SSI), catheter-associated urinary tract infection (CAUTI), hospital-acquired pneumonia (HAP), bloodstream infection (BSI), skin and soft tissue infections (SSTIs), and healthcare-associated Clostridium difficile infection (HCACDI) [5–10]. Hospital-acquired infections represent one of the most common adverse events in healthcare delivery [11].

Hospital-acquired infections may originate from a variety of pathogens, including bacteria, viruses, fungi, and protozoa, which can be transmitted through contact with other hospitalized patients, healthcare personnel, the patient’s own endogenous flora, or contaminated materials. Bacteria account for approximately 90% of HAIs, with gram-negative strains responsible for 51% of these infections [6,9,12]. According to estimates by the World Health Organization (WHO), HAIs affect hundreds of millions of patients globally each year, becoming a major public health concern [8,11,13]. Given their considerable effects on both individual patients and the healthcare system, HAIs necessitate appropriate management.

Globally, it is estimated that between 22% and 73% of treatments for HAIs are inappropriate, despite the WHO’s ongoing efforts to develop and implement policies aimed at promoting the judicious use of antimicrobials [6,14]. Antibiotics are essential, life-saving medicines and are a critical component of universal healthcare [15]. The excessive and misuse of antibiotics are commonly associated with increased rates of adverse drug events, super infections and antimicrobial resistance [16–18]. An expanding body of research suggests that the overuse of medications is a significant and persistent issue in low- and middle-income countries (LMICs) [19]. Inappropriate antimicrobial prescribing is considered to be the leading cause of high burden of AMR in resource-constrained LMICs [20].

The impact of inappropriate treatment of hospital-acquired infections (ITHAIs) is multidimensional [6]. Inappropriate antibiotic therapy (IAT) is associated with higher rates of treatment failure, prolonged hospitalization, and increased mortality, all of which contribute to greater healthcare costs [21]. Each year, HAIs incur societal costs exceeding $200 billion [22]. Inappropriate antimicrobial use—such as insufficient duration, inadequate dosage, or incorrect indication—facilitates the emergence of antibiotic-resistant bacteria, reducing the effectiveness of current therapies [16,17,23,24]. Conversely, the rigorous and timely management of HAIs can reduce patient morbidity and mortality, shorten hospital stays, and lower healthcare expenditures, thereby improving overall patient outcomes and strengthening the healthcare system [25–27].

While previous studies have highlighted the global burden of inappropriate antimicrobial use, there is a notable lack of data regarding the prevalence and underlying factors of inappropriate HAI treatment in resource-constrained regions, including Southern Ethiopia. This study, therefore, aimed to determine the prevalence of inappropriate treatment of HAIs and identify associated factors among admitted adults in Wolaita Zone hospitals, Southern Ethiopia. The findings of this study will contribute to the development of strategies aimed at optimizing the management of HAIs in resource-constrained settings.

## Materials and methods

### Study setting

Wolaita Zone is part of the South Ethiopia Regional State (SERS), Ethiopia. Wolaita is bordered on the south by Gamo Zone, on the west by the Omo River which separates it from Dawro, on the northwest by Kembata Zone and Tembaro Special Woreda, on the north by Hadiya, on the northeast by the Oromia Region, on the east by the Bilate River which separates it from Sidama Region, and on the southeast by Lake Abaya which separates it from Oromia Region. The administrative centre of Wolaita is Wolaita Sodo. It is approximately 327 km from Addis Ababa, the capital city of Ethiopia. According to the 2007 Census conducted by the Central Statistical Agency (CSA) of Ethiopia, the Zone has a total population of 1,501,112 and covers an area of 4,208.64 square kilometers. Wolaita Zone is situated at a latitude of 6°54’N and a longitude of 37°45’E, with an average elevation of 1,850 meters above sea level. According to data from the Wolaita Zone Health Department, the Zone is served by a total of fifteen hospitals. These include one public comprehensive specialized hospital, four private primary hospitals, one general hospital operated by a non-governmental organization (NGO), one NGO-run primary hospital, and eight public primary hospitals. One NGO-run hospital, Soddo Christian General Hospital, was excluded from the study due to its policy prohibiting research activities within the facility.

### Study design and period

A multi-center cross-sectional study was conducted among adult patients with hospital-acquired infections at selected hospitals in the Wolaita Zone from 28 October 2024–25 February 2025.

### Population

The source population comprised all adult patients admitted to hospitals in the Wolaita Zone, Southern Ethiopia, who were diagnosed with hospital-acquired infections. The study population consisted of adult patients aged 18 years and older who were diagnosed with hospital-acquired infections and consecutively selected during the study period. Adult patients diagnosed with hospital-acquired infections who had been admitted for more than 48 hours were included in the study, while those with incomplete medical records were excluded.

### Sample size determination

The sample size was calculated using a single-population proportion formula, based on the following assumption: a prevalence (p) of 66.67% for inappropriate treatment among patients with hospital-acquired infections from a previous study conducted at Zewditu Memorial Hospital, Addis Ababa, Ethiopia [28], 95% confidence level, 5% degree of precision, and z-value at 95% confidence level of 1.96. The Health Management Information System (HMIS) records from each hospital showed that, in the three months before this study, a total of 967 patients had HAIs. The finite population correction formula was applied, as the total population was less than 10,000. Based on the above considerations, the calculated sample size was 252. After accounting for a 10% non-response rate, the final sample size determined for this study was 280.

### Sampling procedure

**Fig 1:**
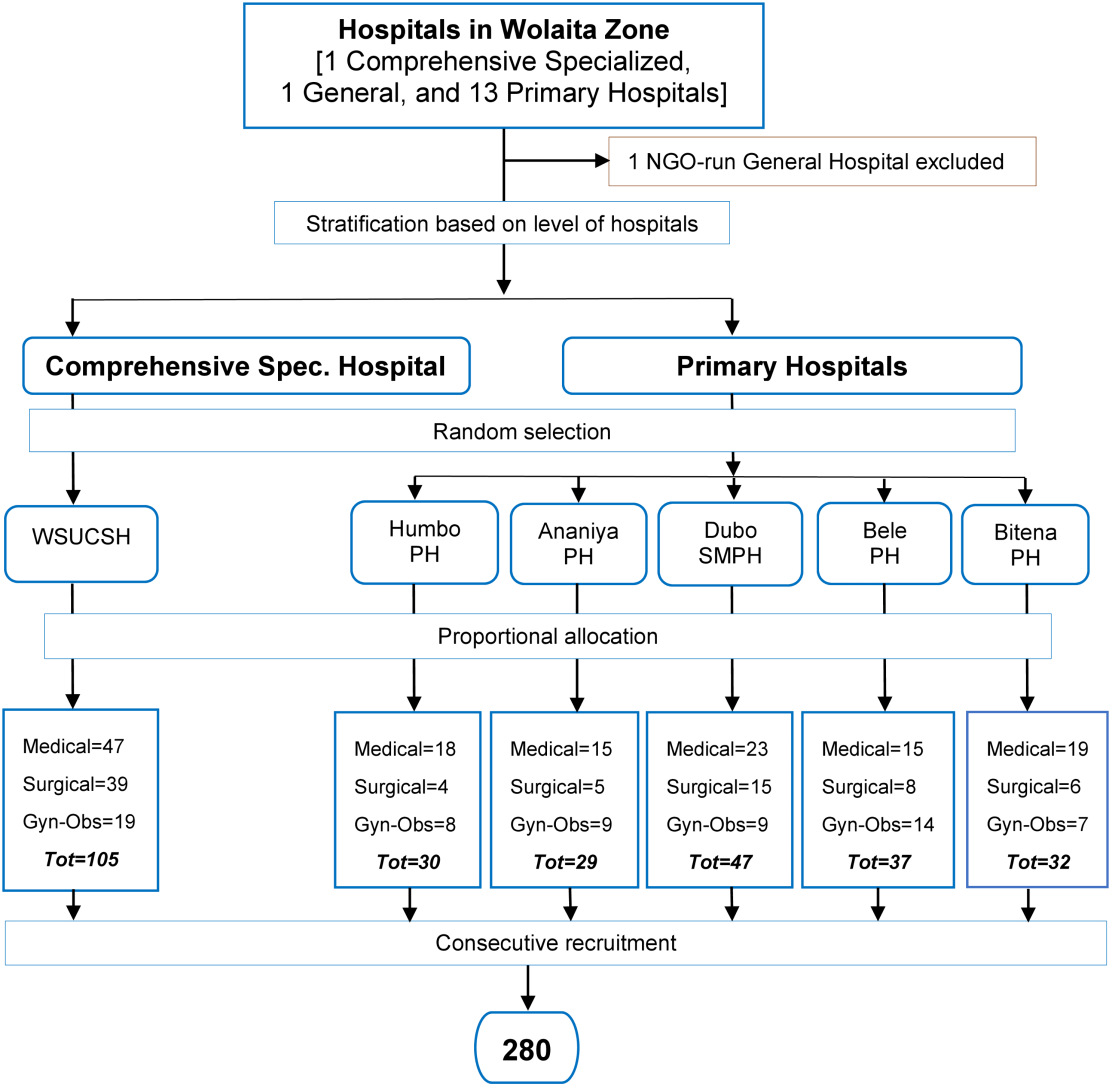
Schematic representation of sampling procedure.

Hospitals were stratified by level of service into two categories: primary hospitals and comprehensive specialized hospitals. Out of the total fourteen hospitals, six were selected for the study. Wolaita Sodo University Comprehensive Specialized Hospital was selected by default, as it was the only hospital in its stratum. Of the thirteen primary hospitals, five were selected based on simple random sampling. The sample size was allocated to each facility based on a proportionate size allocation, using data from the previous three months on patients with hospital-acquired infections in each of the selected hospitals. Accordingly, the sample sizes for the respective hospitals were as follows: Wolaita Sodo University Comprehensive Specialized Hospital – 105 patients, Humbo Primary Hospital – 30 patients, Ananiya Primary Hospital – 29 patients, Dubo Saint Mary Hospital – 47 patients, Bele Primary Hospital – 37 patients, and Bitena Primary Hospital – 32 patients. Patients were consecutively included until the required sample size was achieved, from 28 October 2024–25 February 2025, based on the established eligibility criteria (Fig 1).

### Study variables

#### Dependent variable.

Inappropriate treatment of HAIs was an outcome variable for this study.

#### Independent variables.

The independent variables in this study include socio-demographic, clinical, medication-related (antibiotics and non-antibiotics), investigation-related, healthcare provider-related, and healthcare facility-related factors.

### Operational definitions

#### Measurement of inappropriate treatment:

Inappropriate treatment of HAIs was evaluated based on the most recent edition of the Ethiopian Standard Treatment Guidelines (STG) and the Infectious Diseases Society of America (IDSA) guidelines, with a focus on medication selection, dosage form, frequency and route of administration, potential drug interactions, and duration of therapy. Potential drug interactions were evaluated using the Medscape drug interaction checker. Patients were followed until the completion of treatment for HAIs. Based on the collected data, the treatment of hospital-acquired infections (HAIs) was classified as appropriate or inappropriate according to established criteria. In this study, patients were classified as ‘**Yes**’ (received inappropriate treatment) if at least one deviation from the Ethiopian STG or IDSA criteria was identified; otherwise, they were classified as ‘**No**’ (received appropriate treatment) [20,28–31].

**Catheter- associated urinary tract infection (CAUTI):** a urinary tract infection that occurs with a urinary catheter in place or within 7 days of its placement [9].

**Central line-associated bloodstream infection (CLABSI)**: a serious infection like meningitis and sepsis that occur when bacteria enter the bloodstream through the central line [9].

**Dosage regimen**: refers to the frequency of administration or the number of times the dose is to be taken in a period of time [32].

**Drug interaction**: interaction of drugs or antibiotics prescribed for treatment of HAIs and other drugs a patient is using for treatment of other medical condition. It involves interaction between drugs used for treatment of HAIs if more than one drug is used [33].

**Drug(s) selection**: selecting appropriate drug to treat HAI based on indication, effectiveness, drug-drug interaction, drug-disease interaction and susceptibility profile if culture is done.

**Hospital-acquired infections**: HAIs are illnesses, either localized or systemic, developing after 48 hours of hospitalization as a reaction to an infectious agent, bacteria, or its toxins that were present in hospital settings but were not incubating or exhibiting symptoms when the patient was admitted to the hospital [1, 2].

**Invasive medical devices**: devices inserted either through a body orifice or through the surface of the body and stay in the body during treatment of HAIs. Examples of these include intravenous devices, catheters, Naso-gastric tube [34].

**Prior antibiotics use**: antibiotics used within 90 days before beginning treatment of HAI.

**Length of hospital stay**: the number of days lapsed from diagnosis to completion of treatment of HAIs.

**Surgical site infection**: is a proliferation of pathogenic microorganisms, bacteria, which develops in an incision site within 30 days of operation or within one year if implant is placed [35,36].

**Patients’ clinical outcome status**: these are short term outcomes of treatment of hospital-acquired infections and may be resolved, unimproved, worsening, death and failure.

### Data collection tools and procedures

The data collection tool consists of two components: a structured, interviewer-administered questionnaire used to gather background patient characteristics, and a data abstraction checklist employed to extract relevant information from patients’ medical records, including clinical characteristics, laboratory and other investigations, treatment regimens, healthcare provider-related factors, and health facility-related factors. Six pharmacists, one assigned to each facility, served as data collectors, while an additional pharmacist was appointed to supervise the data collection process.

### Data quality management

The patient interview questionnaire was originally prepared in English and subsequently translated into the local language by a language expert. The principal investigator conducted a two-day training for the data collectors and the supervisor, focusing on the study objectives, the content of the questionnaire, and the procedures for extracting relevant information from medical records. To ensure the reliability of the data collection tools, a pretest was conducted on 5% of the total sample size at Grace Primary Hospital and Kindo Didaye Primary Hospital. Based on the results of the pretest, appropriate modifications were made to the questionnaire. The collected data were reviewed daily to ensure completeness and consistency. In addition, the principal investigator and the supervisor provided daily feedback and corrections to the data collectors. All data were securely stored in a safe and protected location.

### Data processing and analysis

The data were entered using EpiData version 4.6.0 and analyzed using the Statistical Package for the Social Sciences (SPSS) version 25. The data were cleaned to address inconsistencies, then encoded and recoded to ensure appropriate formatting for statistical analysis. Descriptive statistics, including frequency, percentage, mean, standard deviation, median, and interquartile range, were used to examine data distribution and provide a comprehensive summary. A binary logistic regression model was employed to assess the association between inappropriate treatment of HAIs and the explanatory variables. Bivariable logistic regression analysis was initially conducted to identify candidate variables with a p-value < 0.20 for inclusion in the multivariable analysis. In the multivariable logistic regression, variables with a p-value < 0.05 were considered statistically significant independent predictors. The results are presented as crude and adjusted odds ratios, accompanied by their corresponding 95% confidence intervals. Multicollinearity was assessed using collinearity diagnostic statistics, including variance inflation factor (VIF) and tolerance tests. The goodness-of-fit of the model was checked using the Hosmer-Lemeshow test.

### Ethics statement

This research received approval from the Institutional Review Board of the College of Health Sciences and Medicine at Wolaita Sodo University with a reference number: CHSM/ERC/48/17. All procedures adhered to the ethical guidelines outlined in the Declaration of Helsinki for human research. Written informed consent was obtained from all participants prior to their inclusion in the study. All data were collected anonymously, kept confidential, and securely stored during and after the research.

## Results

### Background characteristics of participants

#### Socio-demographic characteristics.

A total of 280 patients who met the eligibility criteria from six hospitals were included in the study. Approximately half of the participants (n = 137, 48.9%) were admitted to the medical ward. Of the participants, 159 (56.8%) were male and 146 (52.1%) were from urban areas. The mean (±SD) age of participants was 41.25 (±12.5) years. The majority (n = 224, 80%) were married. The mean (±SD) monthly income was ETB 5,558.00 (±2,480.50). More than half of the participants (n = 168, 60.0%) were beneficiaries of community-based health insurance (CBHI), while over one-third (n = 113, 40.5%) had completed education up to grades 1–8. The mean duration from hospital admission to the development of HAI was 3.50 (±1.05) days (Table 1).

**Table 1:** Socio-demographic characteristics of adult patients with HAIs in Wolaita Zone hospitals, Southern Ethiopia, from 28 October 2024 to 25 February 2025 (n = 280).

Variables	Frequency	Percent (%)
Age (in years)		
≤ 24	14	5.0
25–34	57	20.3
35–44	87	31.1
45–54	43	15.4
55–64	27	9.6
≥ 65	52	18.6
Sex		
Male	159	56.8
Female	121	43.2
Residence		
Urban	146	52.1
Rural	134	47.9
Educational status		
Unable to read and write	83	29.6
Grade 1–8	113	40.4
Grade 9–12	22	9.9
Higher education	62	22.1
Marital status		
Married	224	80.0
Divorced	11	3.93
Widowed	20	7.14
Single	25	8.93
CBHI beneficiary		
Yes	168	60.0
No	112	40.0
Treatment ward		
Medical	137	48.9
Surgical	77	27.5
Obstetrics and Gynecology (Ob/Gyn)	66	23.6

#### Clinical characteristics.

**Fig 2:**
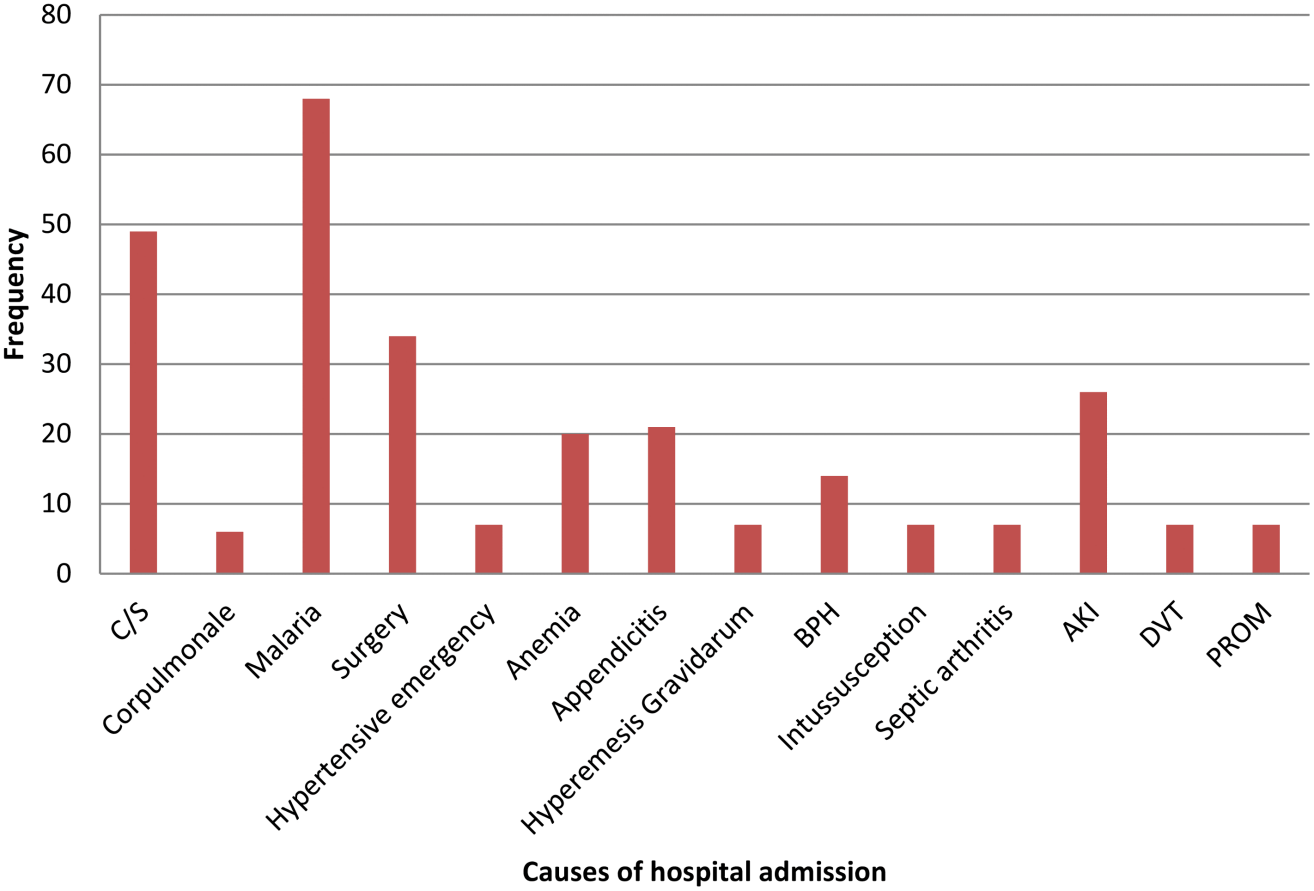
Causes of hospital admission prior to the development of HAI.

Malaria constituted the leading cause of hospital admission, affecting 68 (24.3%) patients, followed by admissions due to cesarean section (n = 49, 17.5%) and other surgical procedures (n = 34, 12.1%) (Fig 2). Additionally, 41 (14.6%) patients had a history of hospital admission within the 90 days preceding their HAI diagnosis.

Almost all participants (n = 277, 98.9%) used invasive medical devices, with peripheral IV lines being the most commonly used (n = 228, 82.3%) (Table 2).

**Table 2:** Types of invasive medical device used.

No.	Invasive devices	Frequency	Percent (%)
1.	Peripheral IV line	228	81.4
2.	Urinary catheter	7	2.5
3.	Peripheral IV line + Urinary Catheter	28	10.0
4.	Central + peripheral IV line inserted	14	5.0

**Fig 3:**
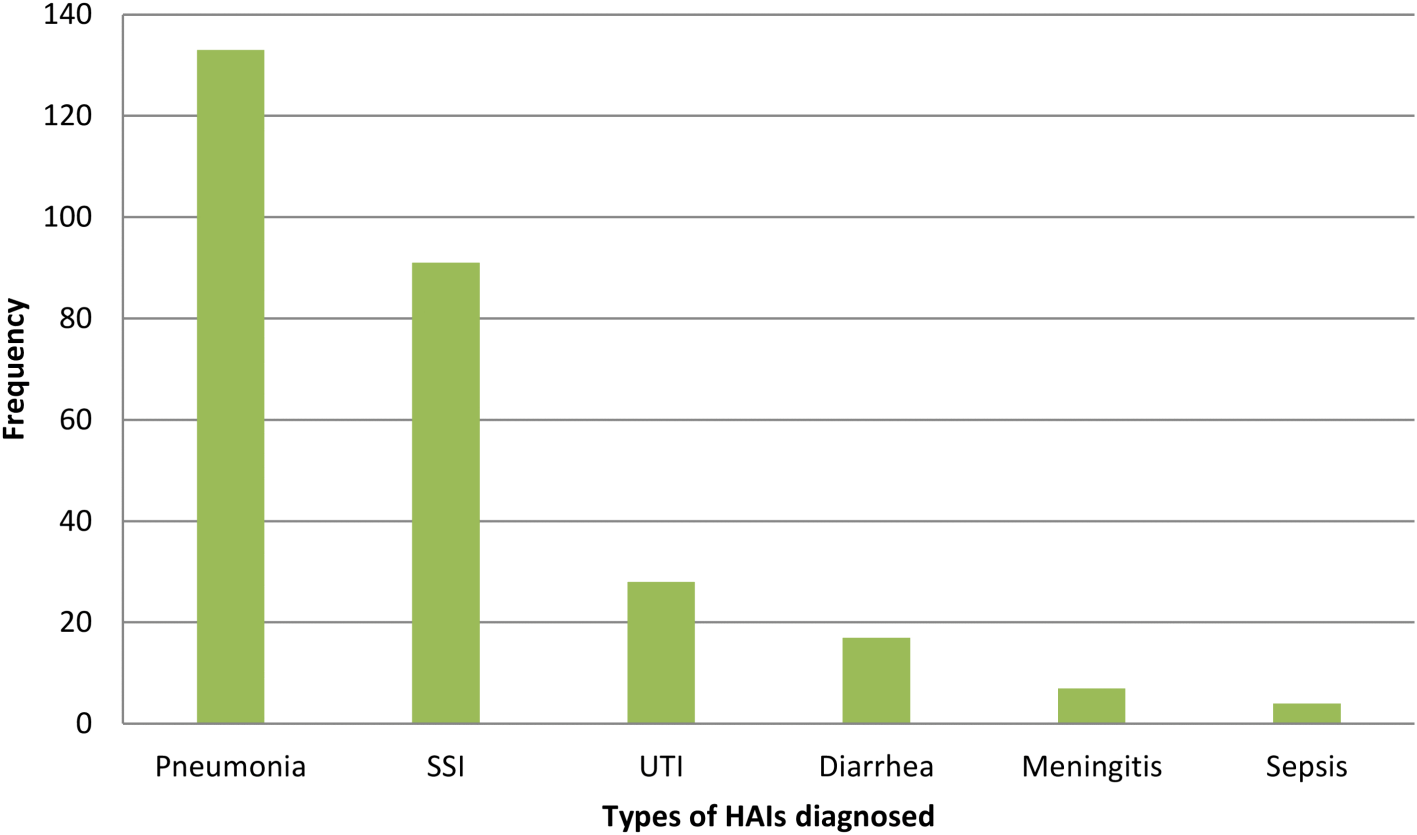
Types of hospital-acquired infections diagnosed and treated among participants.

Pneumonia was the most frequently diagnosed hospital-acquired infection, accounting for 133 (47.5%) cases, followed by surgical site infections (n = 91, 32.5%), urinary tract infections (n = 28, 10.0%), diarrhea (n = 17, 6.1%), meningitis (n = 7, 2.5%), and sepsis (n = 4, 1.4%) (Fig 3).

Among the participants, 122 (43.6%) had comorbid conditions. Diabetes mellitus was the most common, reported in 30 (24.6%) participants, followed by hypertension (n = 27, 22.1%) and anemia (n = 24, 19.7%) (Table 3).

**Table 3:** Comorbid conditions among participants.

No.	Comorbid conditions	Frequency	Percent (%)
1.	Anemia	24	19.7
2.	DM	30	24.6
3.	Hypertension	27	22.1
4.	Cardiac Disease	12	9.8
5.	DVT	12	9.8
6.	BPH	6	4.9
7.	Stroke	4	3.3
8.	Other (Hepatitis, COPD, Asthma...)	7	5.7

#### Investigations and microbiological-related factors.

Laboratory investigations, including CBC, RFT, LFT, BF, INR, and PTT, were performed for 262 (93.6%) participants. Of these, 161 (61.5%) underwent CBC, RFT, and LFT, while BF was conducted for only 17 (6.5%). Imaging investigations such as chest X-ray, ECG, echocardiography (ECHO), and ultrasound were performed for 147 (52.5%) participants. Among these, 52 (35.4%) participants underwent all four imaging modalities (Table 4).

**Table 4:** Laboratory investigations, culture and imaging.

Types of investigations	Frequency	Percent (%)
**Lab investigation performed (n = 280)**		
Yes	262	93.6
No	18	6.4
Types of lab investigation performed (n = 262)		
CBC + RFT + LFT	161	61.5
CBC	56	21.4
CBC + RFT	26	9.9
BF	17	6.5
Others (INR, PTT)	2	0.8
**Culture performed (n = 280)**		
No	211	75.4
Yes	69	24.6
**Imaging performed (n = 280)**		
Yes	147	52.5
No	133	47.5
Types of imaging performed (n = 147)		
Chest X-ray + ECG + ECHO + Ultrasound	52	35.4
Ultrasound	75	51.0
Chest X-ray	20	13.6

Culture results were available for 69 (24.9%) participants. Among these, 14 (20.3%) participants showed no microbial growth, while *Streptococcus* species, *Staphylococcus aureus*, and *Klebsiella pneumoniae* were each isolated in 12 (17.4%) participants (Table 5).

**Table 5:** Pathogens identified and anti-microbial susceptibility test result.

Pathogens identified (n = 69)	Frequency (%)	Susceptible to:	Non susceptible to:
No growth of pathogen	14 (20.3)		
*Streptococcus p*	12 (17.4)	Ampicillin, Chloramphenicol, Ceftriaxone, Ciprofloxacin	
*Klebsiella p*	12 (17.4)	Nalidixic Acid, Chloramphenicol, Ciprofloxacin	Ampicillin, Gentamycin, Ceftriaxone, Tetracycline
*S. Aureus*	12 (17.4)	Ceftriaxone, Nalidixic acid, Cotrimoxazole	Gentamycin, Cloxacillin
*Pseudomonas Aeruginosa*	6 (8.7)	Chloramphenicol, Ampicillin, Ciprofloxacin, Tetracycline, Erythromycin, Augmentin	Ceftriaxone, Clindamycin
*Acinetobacter sp.*	6 (8.7)	Chloramphenicol, Ceftriaxone, Ciprofloxacin, Augmentin	Ampicillin, Cloxacillin
*Citrobacter*	5 (7.2)	Gentamycin, Ceftriaxone, Ciprofloxacin, Clindamycin, Cotrimoxazole	Chloramphenicol, Cloxacillin, Tetracycline, Erythromycin
*E. Coli*	1 (1.4)	Chloramphenicol, Cloxacillin, Erythromycin	Ampicillin, Ceftriaxone, Ciprofloxacin, Cotrimoxazole
*Enterobacter sp.*	1 (1.4)	Gentamycin	Chloramphenicol, Cloxacillin, Ceftriaxone, Nalidixic acid, Ciprofloxacin

#### Utilization of antibiotics and non-antibiotics before and during treatment.

Among the total participants, 93 (33.2%) had received antibiotics within 90 days prior to the treatment of HAI, while 210 (75.0%) were receiving non-antibiotic medications during the treatment of HAI. The most common antibiotic regimen used before HAI diagnosis was Ceftriaxone 1 g IV stat 30 minutes before surgery (n = 72, 77.4%), followed by Cloxacillin 500 mg IV QID for 10 days (n = 7, 7.5%). Ferrous sulfate 325 mg PO TID was the most frequently used non-antibiotic regimen (n = 38, 18%) (Table 6). All participants received treatment on the day of HAI diagnosis. Of these, 35 (12.5%) were treated with Ceftazidime 2g IV BID + Vancomycin 1g IV BID + Ciprofloxacin 400 mg IV BID for 7 days, which is the same number as those who received Ceftazidime 2g IV TID + Vancomycin 1g IV BID for 7 days (S1 Table).

**Table 6:** Utilization of antibiotics and non-antibiotics before and during treatment.

Antibiotics and Non-antibiotics utilization	Frequency (%)
**Antibiotics used prior to treatment of HAI**	
Antibiotics used prior to treatment of HAI (n = 280)	
No	187 (66.8)
Yes	93 (33.2%)
The regimen of antibiotics used prior to treatment of HAI (n = 93)	
Ceftriaxone 2g IV BID for 10 days + vancomycin 1g IV BID for 10 days	4 (4.3)
Ceftriaxone 1g IV stat 30 minutes prior to surgery	72 (77.4)
Cloxacillin 500 mg IV QID for 10days	7 (7.5)
(Ceftriaxone 1g IV BID + Metronidazole 500 mg IV TID + Doxycycline 100 mg PO BID) for 07 days	3 (3.2)
Ceftriaxone 1g IV BID for 07 days + Azithromycin 500 mg daily for 05 days	5 (5.4)
Amoxicillin Clavulanate 625 mg PO TID for 10 days	2 (2.2)
**Non-antibiotics use**	
Non-antibiotics currently on use during treatment of HAI (n = 280)	
No	70 (25.0)
Yes	210 (75.0)
The regimen of non-antibiotics on use (n = 210)	
Metformin 500 mg PO BID + Glibenclamide 5 mg PO BID	18 (8.5)
Nifedipine 30 mg PO BID	16 (7.6)
UFH + Warfarin + Tramadol	9 (4.3)
Paracetamol 1g PO PRN + Metformin 1g BID	5 (2.4)
Lasix 20 mg + UFH	1 (.5)
Omeprazole 20 mg IV BID	18 (8.5)
Morphine 2.5 mg IV QID + UFH	3 (1.4)
Tramadol 100 mg IV PRN	7 (3.3)
Tramadol 50 mg IV TID + Diclofenac 75 mg/3 ml IV BID	2 (.9)
Salbutamol puff	5 (2.4)
Nifedipine 30 mg PO BID + HCT 25 mg daily	11 (5.2)
Ferrous Sulfate 325 mg PO TID	38 (18.0)
ASA 81 mg daily + Atorvastatin 40 mg	7 (3.3)
Ondansetron 4 mg/d + Dexamethasone 4 mg/d	5 (2.4)
Diclofenac 50 mg + Diphenhydramine 25 mg PO BID	12 (5.7)
NPH	8 (3.8)
Tamsulosin 0.5 mg daily	9 (4.3)
Diclofenac 50 mg PO BID	23 (10.9)
Enalapril + Metoprolol	10 (4.7)
Hydralazine IV	4 (1.9)

### Health system-related factors

#### Prescriber-related factors.

**Fig 4:**
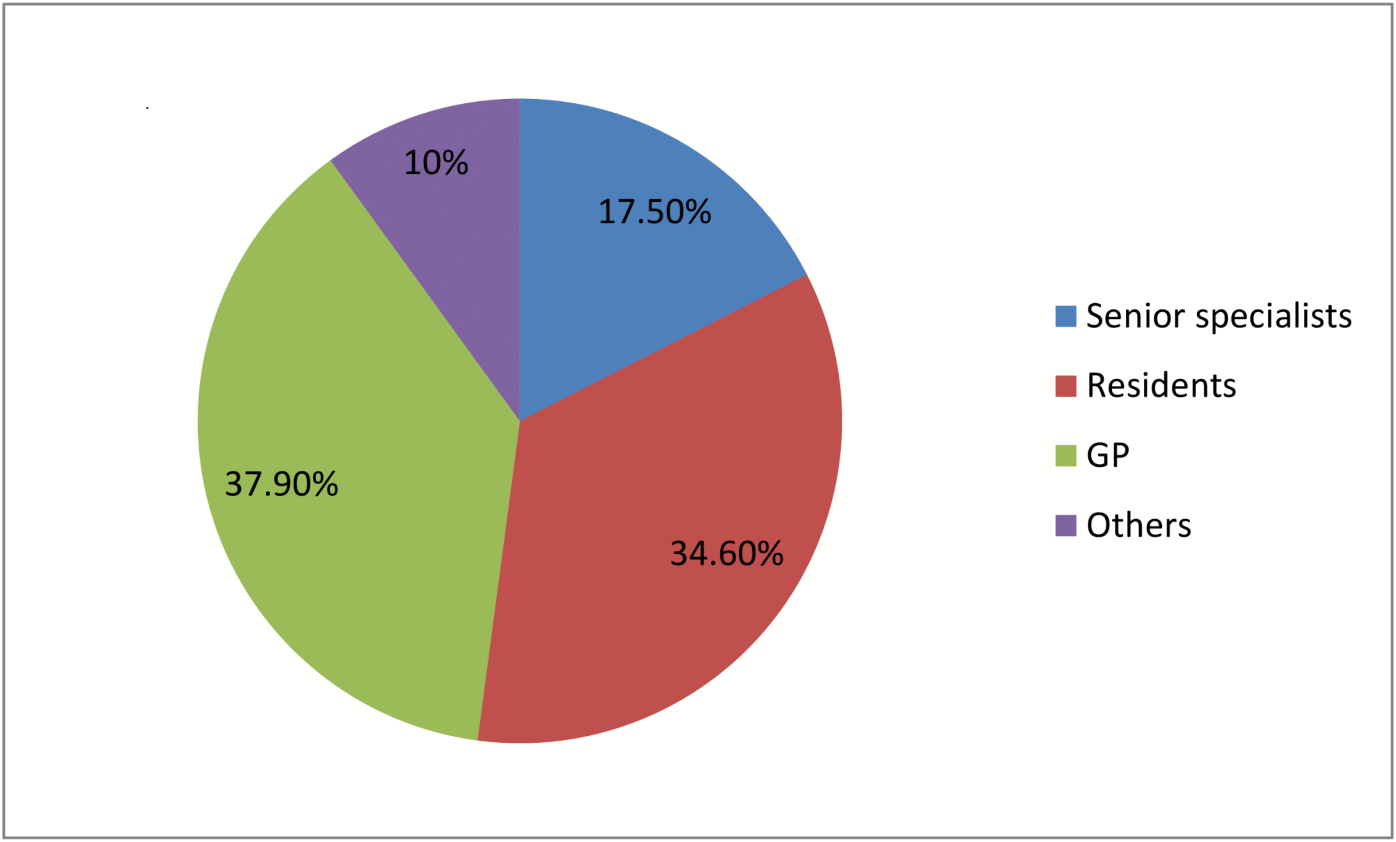
Prescribers’ qualifications.

In the current study, the majority of treatment regimens for HAI were prescribed by general practitioners (n = 106, 37.9%), followed by resident physicians (n = 97, 34.6%) and senior specialists (n = 49, 17.5%) (Fig 4).

#### Health facility-related factors.

**Fig 5:**
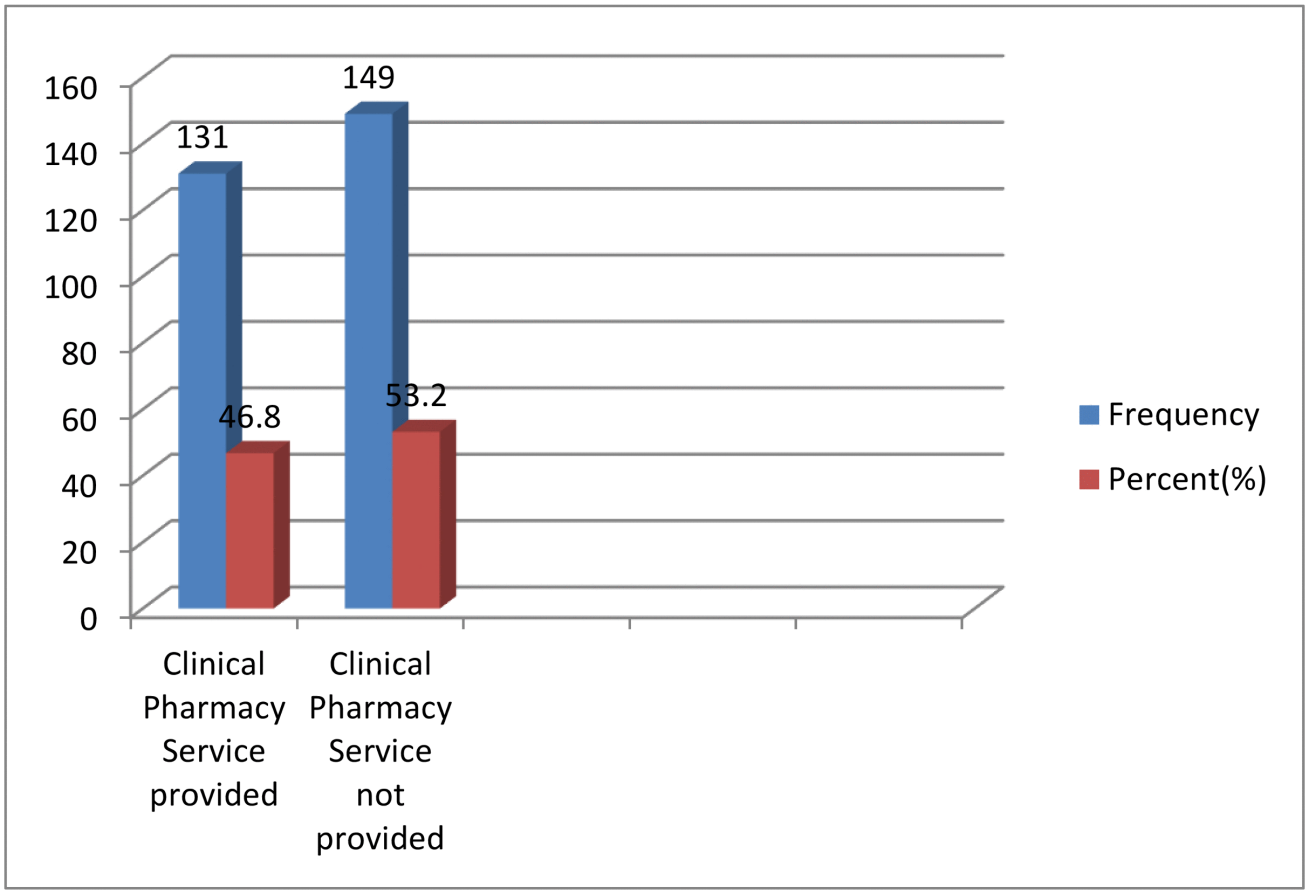
Clinical pharmacy service for treatment of HAIs.

Clinical pharmacy services were provided to 131 (46.8%) participants, and the majority of these services were provided in public hospitals (Fig 5). The majority of participants were from public hospitals (n = 204, 72.9%), and approximately two-thirds (n = 179, 63.9%) were from primary hospitals.

### Prevalence of inappropriate treatment of HAIs

**Fig 6:**
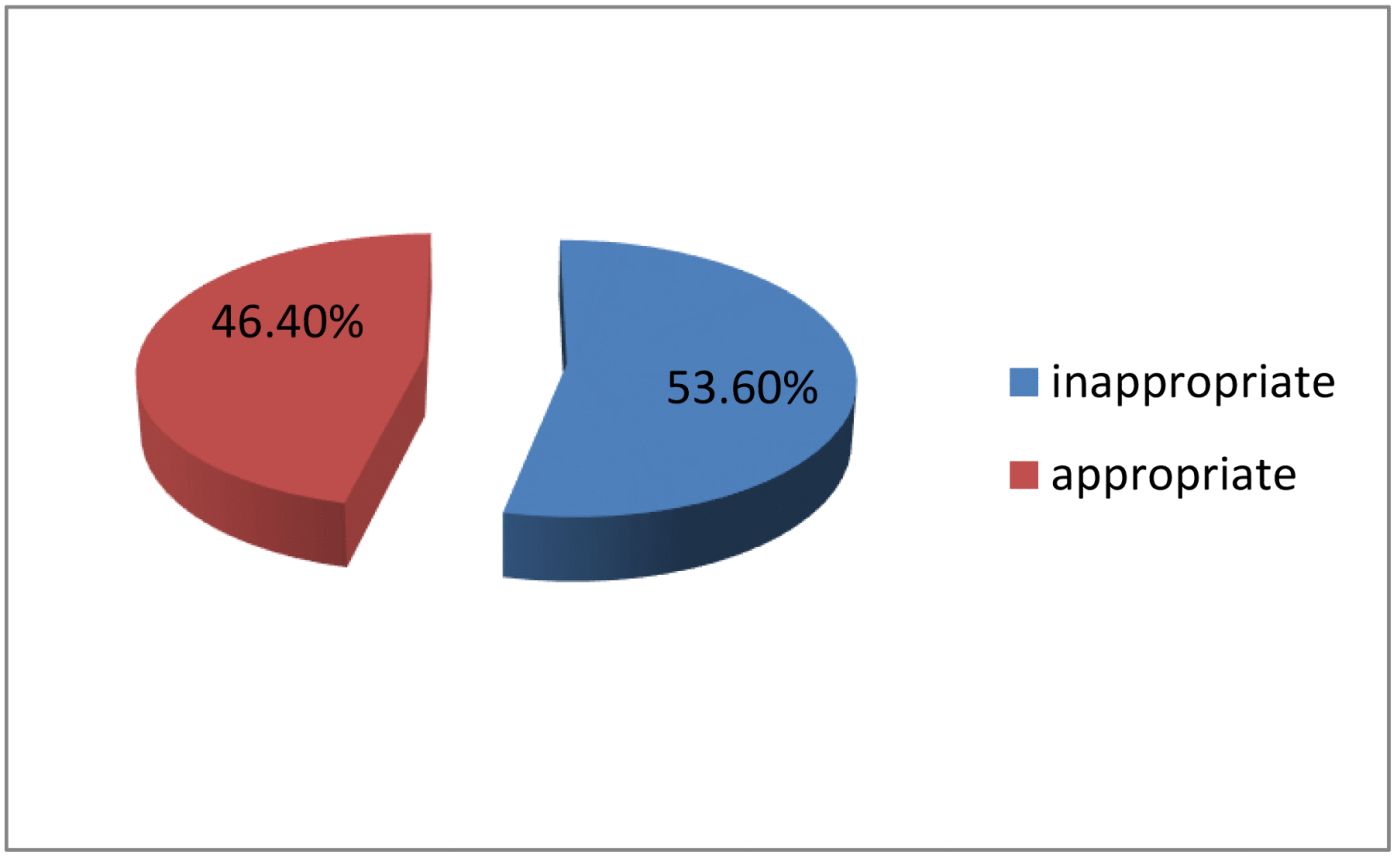
Prevalence of inappropriate treatment of hospital-acquired infections.

**Fig 7:**
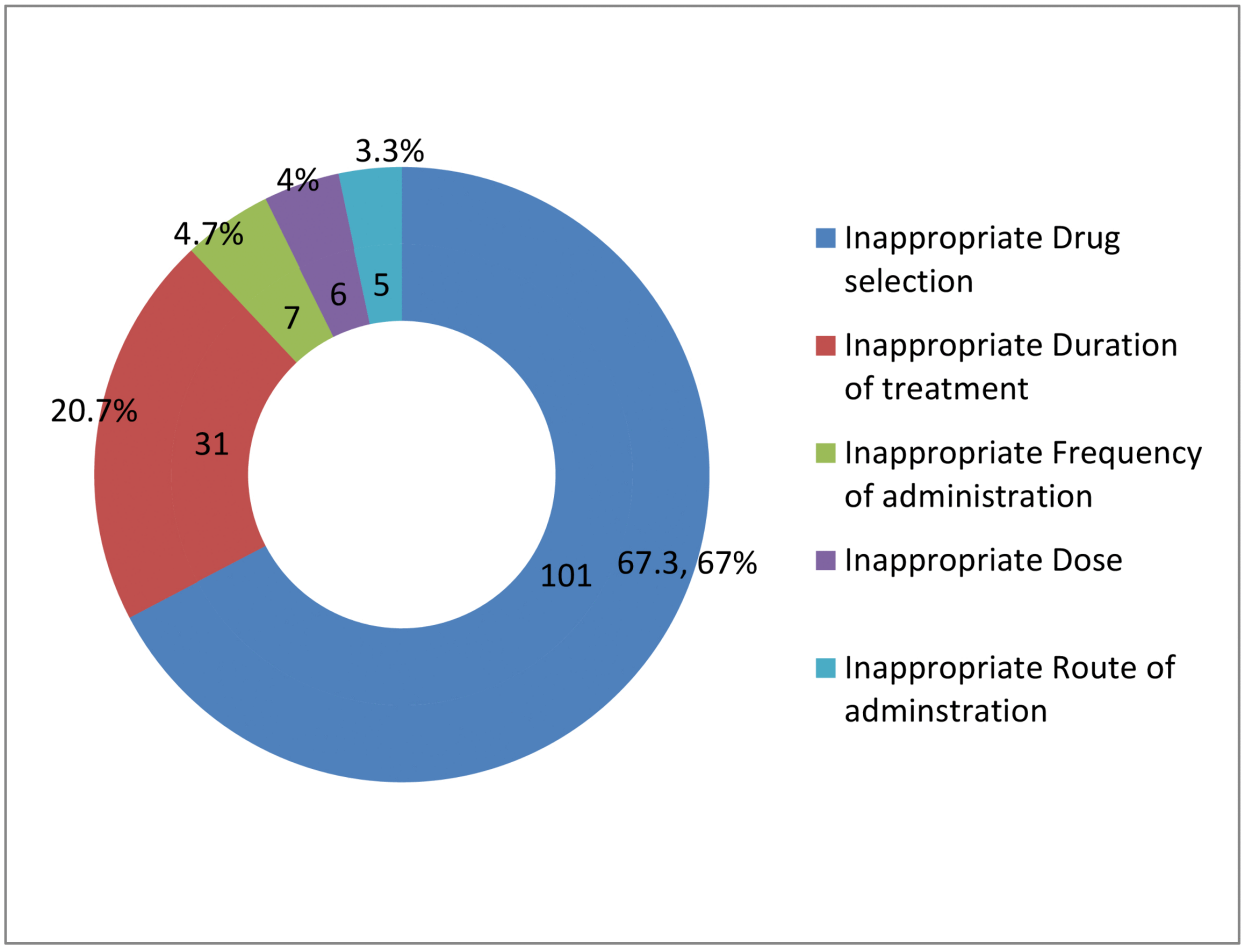
Reasons for inappropriate treatment of hospital-acquired infections.

According to this study, the overall prevalence of inappropriate treatment among adult patients with HAIs was 53.6% (95% CI: 47.5–59.5) (Fig 6). Inappropriate drug selection was the most common reason for the inappropriate treatment of HAIs (n = 101, 67.3%), followed by inappropriate treatment duration (n = 31, 20.7%) (Fig 7).

#### Outcomes of HAIs treatment.

Among the 280 participants who received treatment for HAI, the infection was resolved in 132 (47.1%), while 118 (42.1%) had a poor or only partial improvement. HAI worsened in 25 (8.9%) participants, and 5 (1.8%) died. Of the five patients who died during data collection, 2 (40%) deaths were attributable to HAI, while the remaining 3 (60%) were due to causes other than HAI.

### Factors associated with inappropriate treatment of HAIs

Bivariable logistic regression analysis revealed age, CBHI beneficiary, marital status, presence of comorbidity, imaging performed, culture performed, use of non-antibiotics, provision of clinical pharmacy services, treatment ward, and hospital ownership type as variables with p-values less than 0.2. These variables were subsequently selected as candidates for multivariable logistic regression.

Multivariable logistic regression analysis identified comorbidity, type of treatment ward, culture testing, and the provision of clinical pharmacy services as independent factors associated with inappropriate treatment of hospital-acquired infections among admitted adults in Wolaita Zone hospitals. The odds of receiving inappropriate treatment for HAIs were 2.7 times higher among patients with comorbid conditions compared to those without (AOR = 2.69, 95% CI: 1.45–5.01). Likewise, the odds of receiving inappropriate treatment for HAIs were 2.75 times higher among patients admitted to surgical wards than among those admitted to medical wards (AOR = 2.75, 95% CI: 1.32–5.74). On the other hand, patients who underwent culture testing had 66% lower odds of receiving inappropriate treatment for HAIs compared to those who did not undergo testing (AOR = 0.34, 95% CI: 0.15–0.74). Similarly, patients who received clinical pharmacy services had 72% lower odds of receiving inappropriate treatment for HAIs compared to their counterparts (AOR = 0.28, 95% CI: 0.13–0.60) (Table 7).

**Table 7:** Multivariable logistic regression analysis of factors associated with inappropriate treatment of HAIs among admitted adults in Wolaita Zone hospitals, Southern Ethiopia from 28 October 2024 to 25 February 2025 (n = 280).

Variables	Category	Inappropriate treatment		COR (95% CI)	*p-value*	AOR (95% CI)	*p-value*
		Yes	No				
Age (in years)	≤24	11	3	1		1	
	25–34	33	24	0.36 (0.09–1.49)	0.164	0.99 (0.18–5.51)	0.990
	35–44	39	48	0.22 (0.06–0.85)	0.028	0. 65 (0.10–4.10)	0.646
	45–54	22	21	0.29 (0.07–1.17)	0.082	0.56 (0.09–3.72)	0.548
	55–64	9	18	0.14 (0.03–0.62)	0.01	0.80 (0.10–6.30)	0.828
	≥65	36	16	0.61 (0.15–2.50)	0.496	1.08 (0.16–7.32)	0.938
CBHI beneficiary	No	70	42	1		1	
	Yes	80	88	0.55 (0.34–0.89)	0.015	0.54 (0.30–1.01)	0.053
Marital status	Married	115	109	1		1	
	Widowed	12	8	1.42 (0.56–3.61)	0.459	1.45 (0.41–5.57)	0.570
	Divorced	10	1	9.48 (1.19–75.28)	0.033	2.06 (0.21–20.67)	0.539
	Single	13	12	1.03 (0.45–2.35)	0.950	0. 96 (0.25–3.78)	0.959
Presence of comorbidity	No	61	97	1		1	
	Yes	89	33	4.29 (2.57–7.16)	0.001	**2.69 (1.45–5.01)**	**0.002** ^*^
Imaging performed	No	81	52	1		1	
	Yes	69	78	0.57 (0.35–0.91)	0.02	0.74 (0.38–1.43)	0.367
Culture performed	No	135	76	1		1	
	Yes	15	54	0.16 (0.08–0.30)	0.001	**0.34 (0.15–0.74)**	**0.007** ^*^
Non-antibiotics use	No	27	43	1		1	
	Yes	123	87	2.25 (1.29–3.92)	0.004	1.24 (0.59–2.62)	0.566
Clinical pharmacy service provision	No	105	44	1		1	
	Yes	45	86	0.22 (0.13–0.36)	0.001	**0.28 (0.13–0.60)**	**0.001** ^*^
Treatment ward	Medical	59	78	1		1	
	Surgical	52	25	2.75 (1.53–4.94)	0.001	**2.75 (1.32–5.74)**	**0.007** ^*^
	Ob/Gyn	39	27	1.91 (1.05–3.47)	0.033	2.11 (0.90-4.92)	0.085
Ownership type of hospital	Public	97	107	1		1	
	Private	19	10	2.10 (0.93–4.73)	0.075	0.44 (0.15-1.31)	0.142
	NGO	34	13	2.89 (1.44–5.79)	0.003	0.61 (0.24-1.58)	0.310

* variables statistically significant at *p-value* < 0.05.

## Discussion

This study aimed to assess the prevalence and associated factors of inappropriate treatment of HAIs among admitted adults in Wolaita Zone hospitals, Southern Ethiopia. The prevalence of inappropriate treatment of HAIs was 53.6% (95% CI: 47.5–59.5). The presence of comorbidity, treatment ward type, culture testing, and the provision of clinical pharmacy services were independently associated with the inappropriate treatment of HAIs among admitted adults.

The prevalence of inappropriate treatment of hospital-acquired infections in this study was lower than that observed at Jimma Medical Center, Ethiopia (76.0%) [6], at Zewditu Memorial Hospital in Addis Ababa, Ethiopia (66.67%) [28], and in Lahore, Pakistan (62.8%) [37]. The observed discrepancy in prevalence may be attributed to variations in study settings, sample sizes, study populations (pediatric vs. adult) [28], and clinical practices and healthcare systems across countries [37].

On the other hand, the observed prevalence of inappropriate treatment of HAIs in this study was higher than that reported in a study conducted in Kenyan public hospitals (46.4%) [30] and in a Swiss tertiary care hospital (33.2%) [17]. The observed difference in prevalence may be attributed to variations in the study populations. The study conducted in Kenya enrolled patients from neonatal, pediatric, and adult wards and assessed inappropriate treatment of all infections, whereas our study focused exclusively on hospital-acquired infections (HAIs) among adult patients [30]. The Swiss study enrolled all patients admitted to the University Hospital Basel. Moreover, the hospital has a well-established infectious diseases service that provides consultation upon request, a resource that is lacking in the current study settings [17].

The odds of receiving inappropriate treatment for HAIs were 2.7 times higher among patients with comorbid conditions than among those without. Patients with comorbid conditions are more susceptible to HAIs compared to those without such conditions [38]. In Ethiopia, the presence of underlying non-communicable diseases is a significant risk factor for hospital-acquired infections and may indirectly lead to inappropriate treatment [39]. This finding is consistent with evidence from a study conducted in Tunisia, which reported a significant association between comorbidities and inappropriate antibiotic prescriptions [29]. Similarly, a study conducted in India found that having two or more comorbid conditions was significantly associated with inappropriate antibiotic use [40]. The current finding aligns with a study conducted in Kenya which reported that the presence of multiple diagnoses was associated with increased likelihood of inappropriate treatment [30].

The higher likelihood of inappropriate treatment of HAIs among patients with comorbidities may be attributed to multiple interrelated factors. Managing comorbid conditions often requires complex treatment plans, making it difficult to choose the right medications, dosages, and treatment durations, which can lead to inappropriate treatment. These patients often require multiple medications to manage their underlying conditions, resulting in polypharmacy. Polypharmacy is recognized as the principal risk factor for potentially inappropriate medication use, as well as for adverse drug–drug and drug–disease interactions [41]. Additionally, the presence of comorbidities can extend the duration of hospital stays, thereby increasing the risk of acquiring nosocomial infections.

The odds of receiving inappropriate treatment for HAIs were 2.75 times higher among patients admitted to surgical wards than among those admitted to medical wards. In Ethiopia, patients who undergo surgical procedures are at a higher risk of hospital-acquired infections [39]. In our study setting, approximately one-third (32.5%) of participants developed surgical site infections. This may be one of contributing factors to the administration of inappropriate treatment.

The current finding is supported by evidence from a study conducted at a Swiss tertiary care hospital, which found that admission to a surgical ward was associated with increased odds of inappropriate prescribing [17]. This may be attributed to suboptimal adherence to the surgical antimicrobial prophylaxis protocols outlined in national and international guidelines for the prevention and management of infections in surgical wards. This is supported by a study conducted at Tikur Anbessa Specialized Hospital (TASH), which found that infrequent discussions of surgical antibiotic prophylaxis (SAP) during surgical ward rounds led to practices deviating from established recommendations [42].

Appropriate perioperative antibiotic prophylaxis constitutes a key component of a comprehensive bundle of measures aimed at reducing and preventing postoperative surgical site infections [43–46]. Despite substantial evidence demonstrating improved clinical outcomes and reduced surgical site infections (SSIs) in surgical patients, adherence to these recommendations remains suboptimal across various settings. Multiple studies have reported consistently low compliance with surgical antibiotic prophylaxis [46–56]. The two primary reasons for non-compliance with established guidelines were the inappropriate selection of antibiotics [49,50,53–55] and the improper timing and duration of prophylaxis [49–51,53,55].

Moreover, the limited availability of antibiotics recommended for preoperative prophylaxis and the treatment of surgical site infections in resource-limited settings may impede adherence to established guidelines [51,54,57]. On the other hand, patients in surgical wards who were already receiving antimicrobial therapy for the underlying condition necessitating surgery were frequently prescribed additional, unnecessary preoperative antibiotics. This redundancy may increase the risk of inappropriate treatment of HAIs [58].

Patients who underwent culture testing had 66% lower odds of receiving inappropriate treatment for HAIs compared to those who did not undergo testing. A study conducted at the University of Gondar Specialized Hospital in Ethiopia reported that patients who underwent blood and cerebrospinal fluid culture testing received antibiotics more appropriately than those who did not undergo such testing [59]. Similarly, a study conducted at Jimma Medical Center in Ethiopia found that patients with culture results were less likely to receive inappropriate antimicrobial treatment compared to those without such findings [6].

In the inpatient setting, accurate diagnosis is essential for optimal medication use [60]. Blood cultures are a critical tool used in the diagnosis and management of hospitalized patients [61]. Culture-based diagnostic tests help determine the presence or absence of infection [62]. Although culture testing has the potential to support accurate diagnosis in patients with hospital-acquired infections, only a quarter of patients in our study setting underwent such testing. A low rate of culture testing may hinder effective patient care and contribute to suboptimal medication use [63]. Additionally, underutilization of appropriate diagnostic testing can lead to increased unnecessary empirical antimicrobial use [64,65].

Patients who received clinical pharmacy services had 72% lower odds of receiving inappropriate treatment for HAIs compared to their counterparts. A retrospective cohort study conducted at a tertiary teaching hospital in China on the involvement of clinical pharmacists in the management of central nervous system infections demonstrated that patients in the pharmacy services group had significantly higher scores for the rational use of antibiotics and achieved better improvement rates than the group without pharmacist participation [66]. Similarly, a study on clinical pharmacist-initiated assessment and improvement of appropriate antibiotic use in surgical units at a South Indian tertiary care hospital reported a 95.06% increase in the appropriate use of antibiotics following pharmacist intervention [40]. This may be attributed to the clinical pharmacists’ ability to objectively and comprehensively evaluate medication therapy, enabling timely identification of drug-related problems and appropriate interventions, thereby promoting rational use of medications. Additionally, clinical pharmacists can play a pivotal role in conducting medication reviews and implementing deprescribing interventions, which have been demonstrated to be effective in simplifying therapeutic regimens, minimizing medication-related risks, and reducing potentially inappropriate prescriptions [41,67].

A multidisciplinary team that includes clinical pharmacists is recommended to enhance patient care and mitigate the adverse consequences of antimicrobial overuse or misuse in inpatient settings [68,69]. A recent systematic literature review identified clinical pharmacists as key drivers of effective antimicrobial stewardship interventions, with particular significance in low- and middle-income countries [70]. Clinical pharmacists play a pivotal role in optimizing pharmacotherapy for HAIs through the implementation of diverse, evidence-based strategies. These strategies encompass the correction or minimization of potentially inappropriate medications [71–75], the rationalization of polypharmacy [71,76], the enhancement of antimicrobial prescribing practices [72,77,78], and the reduction of unwarranted antibiotic utilization [72,74,75,79]. Additionally, they can help improve guideline compliance. Evidence suggests that pharmaceutical care interventions significantly improve guideline compliance, which in turn enhances the quality and effectiveness of surgical antimicrobial prophylaxis practices in surgical wards [80–84].

### Implications of the study

The management of HAIs in patients with comorbidities often necessitates complex treatment regimens, warranting careful attention to minimize or prevent inappropriate treatment in this population. Rationalization of polypharmacy should be guided by deprescribing principles and complemented by other evidence-based strategies to ensure optimal therapeutic outcomes.

Surgical patients have a heightened risk of receiving inappropriate treatment; therefore, clinicians responsible for their care should adhere strictly to the established protocols for HAIs outlined in standard guidelines for both preoperative and postoperative periods. Surgeons and other healthcare professionals in surgical wards are encouraged to use antibiotics judiciously in order to maximize clinical cure rates and minimize the emergence of antimicrobial resistance. Combating antibiotic resistance is a collective responsibility of all healthcare professionals [85]. Furthermore, infection control measures should be reinforced to ensure optimal patient outcomes.

Culture testing can play a pivotal role in guiding evidence-based antimicrobial therapy in the inpatient setting. Clinicians are advised to base management decisions, whenever possible, on the results derived from culture testing.

Clinical pharmacists are encouraged to actively contribute to multidisciplinary teams engaged in the management of HAIs [86]. Interactive educational interventions by pharmacists and multidisciplinary quality improvement initiatives could improve surgeons’ adherence to surgical antimicrobial prophylaxis guidelines [87,88]. Their specialized knowledge in pharmacotherapy can play a pivotal role in minimizing polypharmacy and promoting rational antimicrobial prescribing practices, thereby improving patient outcomes [89].

### Strengths and limitations of the study

This multicenter study encompassed a diverse range of hospitals, varying in type and level. Data were collected prospectively, thereby enhancing the quality and reliability of the information compared to retrospective studies. The data collection instrument was developed in accordance with standardized guidelines, ensuring the capture of the most relevant information pertaining to the management of HAIs.

Although the study possesses several strengths, it is not without limitations, and its findings should be interpreted with caution. While the results provide important insights, causal relationships cannot be established. Interpretation of these findings warrants caution. Future studies using more granular hospital-level data or rigorous research designs are needed to better delineate the independent impact of these factors.

Another limitation of this study was its reliance on chart review rather than direct consultation with the treating team, as certain relevant information may not have been documented. Nonetheless, chart review is generally adequate for evaluating many aspects of inappropriate treatment in HAIs. Due to feasibility constraints and limited data availability, the present study did not examine issues related to the cost of treating HAIs.

## Conclusions and recommendations

The prevalence of inappropriate treatment of HAIs among admitted adults in the current study settings was high. Comorbidities, treatment ward type, culture testing, and the provision of clinical pharmacy services were significantly associated with inappropriate treatment.

In the management of HAIs, clinicians should give special consideration to patients with comorbidities, given the complexity of their treatment regimens. For surgical patients, healthcare providers should adhere to established protocols for the management of HAIs as outlined in standard guidelines, both in preoperative and postoperative periods. Hospitals should strengthen microbiological diagnostic services to facilitate evidence-based treatment. Improving clinical pharmacy services in hospital wards is crucial for supporting efforts to minimize inappropriate medication use in managing hospital-acquired infections.

The high prevalence of inappropriate treatment of HAIs underscores the urgent need for comprehensive, multifaceted interventions to improve patient care and combat antimicrobial resistance in this setting.

## Supporting information

S1 Table Treatment status of hospital acquired infection (antimicrobial therapy of HAI) (n = 280). (DOCX)

S1 File Data collection tool English version. (DOCX)

S2 File STROBE-checklist-v4-combined-PlosMedicine. (DOCX)

## Abbreviations

ADR: Adverse Drug Reaction
AMR: Antimicrobial Resistance
AMT: Antimicrobial Therapy
BSI: Blood Stream Infection
CAUTI: Catheter-Associated Urinary Tract Infection
CBHI: Community Based Health Insurance
CDC: Centers for Disease Control and Prevention
CLABSI: A Central Line-Associated Bloodstream Infection
HAI: Hospital-Acquired Infection
HCACD: Health Care Associated Clostridium
IAT: Inappropriate Antimicrobial Therapy
ICU: Intensive Care Unit
ITHAI: Inappropriate treatment of Hospital Acquired Infection
MDR: Multidrug Resistance
SAP: Surgical Antibiotic Prophylaxis
SSI: Surgical Site Infection
SSTI: Skin and Soft Tissue Infections
STG: Standard Treatment Guideline
VAP: Ventilator-Associated Pneumonia
WSUCSH: Wolaita Sodo University Comprehensive Specialized Hospital.
