# Supplementary material for: Inappropriate treatment of hospital-acquired infections and associated factors among admitted adults in Wolaita Zone hospitals, Southern Ethiopia: A multi-center cross-sectional study
Source: PLoS One. 2025 Dec 26;20(12):e0339116. doi: 10.1371/journal.pone.0339116 (PMC12742772; doi:10.1371/journal.pone.0339116)
Supplement: S1 Table — (DOCX) [file pone.0339116.s001.docx]

Supplementary Table 1. Treatment status of hospital acquired infection (antimicrobial therapy of HAI) (n=280).

| **Regimen** | **Frequency (%)** |
| --- | --- |
| Cotrimoxazole 960mg PO BID for 07 days + Metronidazole 500mg PO TID for 07 days | 9 (3.2) |
| Ceftriaxone 1g IV BID for 10 days + azithromycin 500mg po daily for 03 days | 14 (5.0) |
| Ceftazidime 2g IV BID +Vancomycin 1g IV BID + Ciprofloxacin 400mg IV BID for 07 days | 35 (12.5) |
| Gentamycin 80mg IV BID for 05 days + Ceftazidime 2g IV TID | 7 (2.5) |
| ceftriaxone 1g IV BID for 10days + azithromycin 500mg po daily for 03 days | 19 (6.8) |
| Ceftriaxone 1g IV BID for 07 days + Gentamycin 80mg daily for 05 days | 19 (6.8) |
| Ceftriaxone 1g IV BID for 14 days + Azithromycin 500mg PO daily for 05 days | 17 (6.1) |
| Ceftazidime 2g IV BID for 14 days | 4 (1.4) |
| (Ceftazidime 2g IV TID + Vancomycin 1g IV BID), 05days | 11 (3.9) |
| (Ceftriaxone 1g IV BID + Metronidazole 500mg IV TID) for 10days | 31 (11.1) |
| Cephalexin 500mg po BID for 05 days + Metronidazole 500mg PO TID for 03 days | 6 (2.1) |
| Cloxacillin 500mg PO QID for 07 days + Ceftriaxone 1g IV BID for 07 days | 27 (9.6) |
| Metronidazole 500mg IV TID for 07 days | 11 (3.9) |
| (Ceftazidime 2g IV TID + Vancomycin 1g IV BID) for 07 days | 35 (12.5) |
| Ceftriaxone 2g IV BID +Vancomycin 1g IV BID + Ampicillin 500mg PO QID for 21 days | 4 (1.4) |
| Metronidazole 500mg PO TID for 10 days + Ceftriaxone 1g IV BID for 10 days | 5 (1.8) |
| Ceftazidime 2g IV TID for 05 days + Gentamycin 80mg/day for 05 days | 5 (1.8) |
| (Vancomycin 1g IV BID +Ceftazidime 2g IV TID + Metronidazole 500mg IV TID), 07 days | 7 (2.5 |
| Cephalexin 500mg PO BID for 07 days + Meropenem | 10 (3.6) |
| Cefepime 2g IV BID for 07 days + gentamycin 80mg/d for 07 days | 4 (1.4) |
| Changing new regimen | |
| Regimen change done |  |
| No | 253 (90.4%) |
| Yes | 27 (9.6%) |
| Changed regimen (n=27) |  |
| Ceftazidime 2g IV TID for 10 days | 9 (33.3) |
| Ceftazidime 2g IV TID + Vancomycin 1g IV BID for 07 days | 7 (25.9) |
| Gentamycin 80mg daily for 10 days | 4 (14.8) |
| Ciprofloxacin 400mg IV BID for 5 days + Vancomycin 1 g IV BID for 7 days | 1 (3.7) |
| Clindamycin 600mg BID for 7 days | 6 (22.2) |
| Reason for changing regimen (n= 27) | |
| Reason |  |
| After culture result | 7 (25.9%) |
| Due to recommendation by clinical pharmacist | 20 (74.1%) |
| Mean (SD) of antibiotics used per entire treatment of HAI | 2.18 (±0.655) |
| Mean (SD) of time taken to change regimen from time starting initial treatment in days | 3.15 (±1.110) |
| Mean (SD) time taken to complete treatment of HAI in days | 8.95 (±2.052) |
| Mean (SD) time taken to start treatment after diagnosis of HAI in days | 0 (±0) |
