# Supplementary material for: Inappropriate treatment of hospital-acquired infections and associated factors among admitted adults in Wolaita Zone hospitals, Southern Ethiopia: A multi-center cross-sectional study
Source: PLoS One. 2025 Dec 26;20(12):e0339116. doi: 10.1371/journal.pone.0339116 (PMC12742772; doi:10.1371/journal.pone.0339116)
Supplement: S1 File — (DOCX) [file pone.0339116.s002.docx]

**Data Collection Tool English Version**

**Section I: Questionnaire**

Date of data collection________________ Participant’s code __________________

Name of data collector________________________________

| **Sr.No.** | **Questions** | | | | | | **Response** | | | | | | | | |
| --- | --- | --- | --- | --- | --- | --- | --- | --- | --- | --- | --- | --- | --- | --- | --- |
| **10000** | **Section I: data to be gathered by patient interview** | | | | | | | | | | | | | | |
| **11000** | **Sub-section I: Patient related information** | | | | | | | | | | | | | | |
| **11100** | **Part I: Patient’s Socio-demographic information** | | | | | | | | | | | | | | |
| 11101 | Sex | | | | | | 1. Male 2. Female | | | | | | | | |
| 11102 | Age | | | | | | _______ Years | | | | | | | | |
| 11103 | Marital Status | | | | | | 1. Married 2. Widowed  3. Divorced 4.Single | | | | | | | | |
| 11104 | Average Monthly Income | | | | | | ____ ETB | | | | | | | | |
| 11105 | Are you beneficiary of CBHI | | | | | | 1. No 2. Yes | | | | | | | | |
| 11106 | Educational status of participant | | | | | | 1. Not able to read and write 2. Grade 1-8 3. Grade 9-12 4. Higher education(diploma, degree and above) | | | | | | | | |
| 11107 | Residency of the patient | | | | | | 1. Urban 2. Rural | | | | | | | | |
| 11200 | **Part II: Findings on Admission** | | | | | | | | | | | | | | |
| 11201 | Admission date | | | | | |  | | | | | | | | |
| 11202 | Reason for admission | | | | | |  | | | | | | | | |
| 11203 | Previous medical admission | | | | | | 1. Yes 2. No | | | | | | | | |
| 11204 | If yes to question no. 111400, date of admission | | | | | |  | | | | | | | | |
| 11205 | Length of Hospital stay, if yes to question no.111400 | | | | | | from to E.C | | | | | | | | |
| **200000** | **Section II: Data abstraction checklist for medical records** | | | | | | | | | | | | | | |
| **2100000** | **Sub-section I: patient related factors** | | | | | | | | | | | | | | |
| **2110000** | **Part I: Clinical characteristics of HAIs** | | | | | | | | | | | | | | |
| **2111000** | **Clinical presentation** | **Yes (√)/No(x)** | | | | | **No.** | **Vital signs** | | | | | | **Value** | |
|  |  | **At diagnosis of HAI** | **On date of regimen DC of HAI** | | | |  |  | | | | | **At diagnosis of HAI** | | **On date of regimen DC of HAI** |
| 211101 | Fever |  |  | | | | 211110 | Temperature, ℃ | | | | |  | |  |
| 211102 | Cough |  |  | | | | 211111 | BP, mmHg | | | | |  | |  |
| 211103 | Headache |  |  | | | | 211112 | HR | | | | |  | |  |
| 211104 | Pain |  |  | | | | 211113 | RR | | | | |  | |  |
| 211105 | Sweating |  |  | | | | 211114 | O2 sat off/on | | | | |  | |  |
| 211106 | Urine urgency |  |  | | | |  |  | | | | |  | |  |
| 211107 | Frequent urination |  |  | | | |  |  | | | | |  | |  |
| 211108 | Dysuria |  |  | | | |  |  | | | | |  | |  |
| 211109 | Others (specify) |  |  | | | |  |  | | | | |  | |  |
| **212000** | **Part II: Invasive Medical devices used related variables** | | | | | | | | | | | | | | |
| **212100** | **Invasive medical devices used** | | | | | | **Yes** | | | | | | **No.** | | |
| **212200** | **If yes to question no. 212100, mark (**√**) under ʺYesʺ for respective options** | | | | | | | | | | | | | | |
|  | **Variables** | | | | | | **Yes** | | | | | | | | |
| 212201 | Central IV line inserted | | | | | |  | | | | | | | | |
| 212202 | Peripheral intravenous line inserted | | | | | |  | | | | | | | | |
| 212203 | Urinary catheter inserted | | | | | |  | | | | | | | | |
| 212204 | Naso-gastric Tube inserted | | | | | |  | | | | | | | | |
|  | Other ( Specify ) | | | | | |  | | | | | | | | |
| 212205 |  | | | | | |  | | | | | | | | |
| 212206 |  | | | | | |  | | | | | | | | |
| **213000** | **Part III : Hospital acquired infections related variables:** | | | | | | | | | | | | | | |
| **213100** | Date HAIs­­­­­­­­­­­­­­­­­­ was diagnosed. | | | | | | | | | | | | | | |
| **213200** | **Type of HAIs diagnosed; mark ʺ√ʺ if respective disease is diagnosed and ʺХʺ if not** | | | | | | **Yes** | | | | | | | | |
| 213201 | Pneumonia | | | | | |  | | | | | | | | |
| 213202 | Urinary tract infection | | | | | |  | | | | | | | | |
| 213203 | Surgical site infection | | | | | |  | | | | | | | | |
| 213204 | Diarrhea | | | | | |  | | | | | | | | |
| 213205 | Bone and joint infection (specify) | | | | | |  | | | | | | | | |
| 213206 | Blood stream infections (sepsis, meningitis) | | | | | |  | | | | | | | | |
| 213207 | Others (specify)------- | | | | | |  | | | | | | | | |
| **214000** | **Part IV : Co morbidity** | | | | | | | | | | | | | | |
| **214100** | Is there any co morbid condition? | | | | | | Yes | | | | | | No | | |
| **214200** | **If yes to question number 24100, mark ʺ√ʺ in next column of respective disease** | | | | | | | | | | | | | | |
| 214201 | Cardiac disease | | | | | |  | | | | | | | | |
| 214201 | Diabetic mellitus | | | | | |  | | | | | | | | |
| 214203 | Hypertension | | | | | |  | | | | | | | | |
| 214204 | HIV | | | | | |  | | | | | | | | |
| 214205 | TB | | | | | |  | | | | | | | | |
| 214206 | Thyroid disorder | | | | | |  | | | | | | | | |
| 214207 | Benign prostate hyperplasia | | | | | |  | | | | | | | | |
| 214208 | Malignancy | | | | | |  | | | | | | | | |
|  | Others (specify) | | | | | |  | | | | | | | | |
| 214209 |  | | | | | |  | | | | | | | | |
| 214210 |  | | | | | |  | | | | | | | | |
| **220000** | **Sub-section II : Investigations and Medications related data:** | | | | | | | | | | | | | | |
| **221000** | **Part I: Investigations** | | | | | | | | | | | | | | |
| **221100** | **1 : Laboratory results:** | | | | | | | | | | | | | | |
| **2211100** | **Is lab investigation done?** | | | | | **Yes No** | | | | | | | | | |
| **22111100** | **If yes to question no. 2211100, mark ʺ√ʺ in front of respective investigations** | | | | | | | | | | | | | | |
|  | **Investigations** | | | | | **Done** | | | | **Results of Abnormal Value** | | | | | |
| **22111101** | **CBC** | | | | |  | | | |  | | | | | |
| **22111102** | **RFT** | | | | |  | | | |  | | | | | |
| **22111103** | **LFT** | | | | |  | | | |  | | | | | |
|  | **Other lab results(specify)** | | | | |  | | | |  | | | | | |
| **22111104** |  | | | | |  | | | |  | | | | | |
| **22111105** |  | | | | |  | | | |  | | | | | |
| **2212000** | **2: Microbiologic findings** | | | | | | | | | | | | | | |
| **2212100** | **Is culture done?** | | | | | **Yes No** | | | | | | | | | |
| **22121100** | **If yes to question no. 2212100, fill the following table** | | | | | | | | | | | | | | |
|  | **Pathogen identified** | | | **Sensitive To** | | | | | **Resistant To** | | | | | | |
| **22121101** |  | | |  | | | | |  | | | | | | |
| **22121102** |  | | |  | | | | |  | | | | | | |
| **22121103** |  | | |  | | | | |  | | | | | | |
| **2213000** | **3: Imaging finding:** | | | | | | | | | | | | | | |
| **2213100** | **Is imaging done?** | | | | | **Yes No** | | | | | | | | | |
| **2213200** | **If yes to question no. 2213100, mark ʺ√ʺ in front of respective imaging** | | | | | | | | | | | | | | |
|  | **Imaging findings** | | | | | **Done** | | | | | | | | | |
| **2213201** | Chest X-ray | | | | |  | | | | | | | | | |
| **2213202** | ECG | | | | |  | | | | | | | | | |
| **2213203** | Echo | | | | |  | | | | | | | | | |
| **2213204** | Ultrasound | | | | |  | | | | | | | | | |
|  | Others | | | | |  | | | | | | | | | |
| **2213205** |  | | | | |  | | | | | | | | | |
| **2213206** |  | | | | |  | | | | | | | | | |
| **222000** | **Part II: Medication related data:** | | | | | | | | | | | | | | |
| **222100** | **1: Data on prior antibiotics use** | | | | | | | | | | | | | | |
| **2221100** | **Is there any antibiotics used prior to treatment of HAIs? Yes No** | | | | | | | | | | | | | | |
| **2221200** | **If yes to question no. 2221100, Date antibiotics used** | | **From _______________to______________** | | | | | | | | | | | | |
| **2221300** | **If yes to question no. 2221100, fill the following table accordingly.** | | | | | | | | | | | | | | |
|  | **Medications used** | | | | | | **Regimen** | | | | | | | | |
| **2221301** |  | | | | | |  | | | | | | | | |
| **2221302** |  | | | | | |  | | | | | | | | |
| **2221303** |  | | | | | |  | | | | | | | | |
| **2221304** |  | | | | | |  | | | | | | | | |
| 222200 | 2**.Data on non-antibiotics on use** | | | | | | | | | | | | | | |
| 2222100 | **Is there any non-antibiotics on use Yes No** | | | | | | | | | | | | | | |
| 2222200 | **If yes to question no. 2221100, fill the following table accordingly.** | | | | | | | | | | | | | | |
|  | **Medications used** | | | | | | **Regimen** | | | | | | | | |
| 2222201 |  | | | | | |  | | | | | | | | |
| 2222202 |  | | | | | |  | | | | | | | | |
| 2222203 |  | | | | | |  | | | | | | | | |
| 2222204 |  | | | | | |  | | | | | | | | |
| 2222205 |  | | | | | |  | | | | | | | | |
| 222300 | **3. Data on treatment status of patients for HAI:** | | | | | | | | | | | | | | |
| **2223100** | **Date the treatment was started** | | | | | | | | | |  | | | | |
| **2223200** | **Treatment regimen** | | | | | | | | | |  | | | | |
|  | **Antibiotics** | | **Dose, route and frequency** | | | | | | | | **Duration** | | | | |
| **2223201** |  | |  | | | | | | | |  | | | | |
| **2223202** |  | |  | | | | | | | |  | | | | |
| **2223203** |  | |  | | | | | | | |  | | | | |
| **2223204** |  | |  | | | | | | | |  | | | | |
| **2223300** | **Is there any change of regimen? 1. Yes 2. NO, go to question no. 2223600** | | | | | | | | | | | | | | |
| **2223400** | **Date the regimen was changed** | | | | | |  | | | | | | | | |
| **2223500** | **Reason for changing regimen** | | | | | | 1. Treatment failure **2.** After culture **3.** Resistance **4.** Adverse drug reaction **5.** Contraindication **6**. Due to recommendation by clinical pharmacist | | | | | | | | |
| **22235600** | **Changed regimen** | | | | | | | | | | | | | | |
|  | **Antibiotics** | | | | **Dose, route and frequency** | | | | | | | **Duration** | | | |
| **2223601** |  | | | |  | | | | | | |  | | | |
| **2223602** |  | | | |  | | | | | | |  | | | |
| **2223603** |  | | | |  | | | | | | |  | | | |
| **2223604** |  | | | |  | | | | | | |  | | | |
| **2223701** | **Date the treatment was discontinued** | | | | | |  | | | | | | | | |
| **2223702** | **Number of antibiotics per treatment** | | | | | |  | | | | | | | | |
| **2223703** | **Is the initial treatment inappropriate?** | | | | | | **1. Yes 2. No** | | | | | | | | |
| **2223704** | **If yes to question no. 222380, Reason for inappropriate treatment** | | | | | | 1. Indication 2. Dose 3. Frequency 4. Duration 5. Route of Administration 6. Drug-drug Interaction 7. Drug-disease interaction 8. Ineffective | | | | | | | | |
| **2223705** | **Is the changed treatment inappropriate?** | | | | | | **1. Yes 2. No** | | | | | | | | |
| **2223706** | **If yes to question no. 222400, Reason for inappropriate treatment** | | | | | | 1. Indication 2. Dose 3. Frequency 4. Duration 5. Route of Administration 6. Drug-drug Interaction 7. Drug-disease interaction 8. Ineffective | | | | | | | | |
| **230000** | **Sub-section III: Data on health care provider (prescriber)** | | | | | | | | | | | | | | |
| **2301** | **Educational status of the health care provider (prescriber) for initial treatment** | | | | | | 1. Senior specialist 2. Resident 3. General Practitioner 4. Other (Specify________________________) | | | | | | | | |
| **2400** | **Sub-section IV: Health care facility related data** | | | | | | | | | | | | | | |
| **2401** | Is clinical pharmacy service provided? | | | | | | 1. Yes 2. No | | | | | | | | |
| **2402** | Ownership type of hospital | | | | | | 1. Public 2. Private 3.NGO | | | | | | | | |
| **2403** | Level of care of the hospital | | | | | | 1. Comprehensive specialized hospital 2. Primary hospital | | | | | | | | |
| **2404** | Treatment unit | | | | | | 1. Medical ward 2. Surgical ward 3. gyn/obs ward | | | | | | | | |
| **2500** | **Sub-section V: Outcome measurement:** | | | | | | | | | | | | | | |
| 2501 | Inappropriate treatment of HAIs | | | | | | 1. Yes 2. No | | | | | | | | |
| 2502 | If Yes to question No. 2501, The reason for inappropriate treatment of HAI | | | | | | 1. Indication 2. Dose 3. Frequency 4. Duration 5. Route of Administration 6. Drug-drug Interaction 7. Drug-disease interaction 8. Ineffective | | | | | | | | |
| **2600** | **Sub-section VI: Patient outcome** | | | | | | | | | | | | | | |
| 2601 | Total length of hospital stay | | | | | | (from to ) | | | | | | | | |
| 2602 | Clinical Status of the patient | | | | | | 1. Resolved 2. Partially improved 3. Worsened 4. Death. | | | | | | | | |
| 2603 | If option for question no.262200 is death, cause of the death | | | | | | 1. HAI 2. Other than HAI | | | | | | | | |

| **2404** | Treatment unit | 1. Medical ward 2. Surgical ward 3. gyn/obs ward |
| --- | --- | --- |
| **2500** | **Sub-section V: Outcome measurement:** | |
| 2501 | Inappropriate treatment of HAIs | 1. Yes 2. No |
| 2502 | If Yes to question No. 2501, The reason for inappropriate treatment of HAI | 1. Indication 2. Dose 3. Frequency 4. Duration 5. Route of Administration 6. Drug-drug Interaction 7. Drug-disease interaction 8. Ineffective |
| **2600** | **Sub-section VI :Patient outcome** | |
| 2601 | Total length of hospital stay | (from to ) |
| 2602 | Clinical Status of the patient | 1. Resolved 2. Partially improved 3. Worsened 4. Death. |
| 2603 | If option for question no.262200 is death, cause of the death | 1. HAI 2. Other than HAI |
